# Why is it difficult for children and adults to follow a person’s eye gaze in polynomial social relationships with compound audio-visual stimuli: An eye-tracking study

**DOI:** 10.1371/journal.pone.0289404

**Published:** 2023-08-01

**Authors:** Misaki Oka, Mikimasa Omori

**Affiliations:** 1 School of Human Sciences, Waseda University, Tokorozawa, Saitama, Japan; 2 Faculty of Human Sciences, Waseda University, Tokorozawa, Saitama, Japan; The University of Alabama, UNITED STATES

## Abstract

Acquiring triadic social interactions could facilitate language and communication skills in early infancy. However, studies have rarely investigated polynomial relationships, defined as relationships among the self, two or more people, and objects. During the development from a child to an adult, the responsiveness to a preferred stimulus modality changes from visual to auditory dominance. Nevertheless, how people observe compound visual stimuli in polynomial social relationships and why it is difficult to ignore auditory cues remain unclear. Moreover, there is a need to identify differences between children’s and adults’ observing latencies in the time to the first fixation when detecting a stimulus. This study examined whether participants (24 adults and 19 children) demonstrated similar gaze patterns under triadic and polyadic conditions. The participants observed a target visual stimulus looked at by a face stimulus while we presented spoken names, either congruent or incongruent with the target visual stimulus. The results indicated that when the number of people in social relationships increased, children and adults decreased fixations on the target face and the stimulus and showed a shorter mean fixation duration on the face. Moreover, children had longer latencies and more fixation errors for the target stimulus, which might reflect children’s difficulties in communicating with others. We expect that understanding children’s communication transition from triadic to polynomial social relationships with audio-visual stimulus congruencies would facilitate understanding language development and social communication patterns.

## Introduction

Understanding what others look at is essential for living in social environments. From early infancy, humans directly acquire information in dyadic social relationships through eye-to-eye contact with others. At approximately nine months of age, we learn to follow another person’s gaze or their finger pointing at an object in triadic social relationships leading to language and social communication development [1,2]. A recent study of triadic social relationships using an eye tracker [3] demonstrated that typically developing adults have longer saccade latencies and make more saccade direction errors when following a person’s gaze if a visual cue is inconsistent with where a face stimulus is looking than when it is consistent.

The significance of developing triadic relationships is well-known, and several studies have evaluated relationships among the self, another person, and an object [1–3]. However, researchers have rarely investigated polynomial relationships consisting of the self, more than two other people, and objects [4]. Bristow and colleagues [4] showed that when a person looks directly at the other’s face, typically developing adults detect the gaze location faster after the person’s gaze shifts towards the other’s face than when the person is not looking at the other person. These results [3,4] suggest that adult participants took longer to detect the eye gaze location when presenting more than two people Moreover, presenting multiple people distracts opportunities for following a person’s gaze because we must ignore the visual cues presented by unexpected people. However, one of the above studies [3] mainly focused on saccades and used a colored dot as a visual cue for gaze following. The other [4] study examined brain functions when detecting gaze locations in two people but did not collect eye-movement data. However, focusing on eye movements, fixations, and dwelling patterns is necessary to examine the gaze-following behavior in polynomial social relationships in terms of analyzing the shifting process of polynomial social relations from triadic relations.

Only a few studies [5,6] have investigated eye-movement patterns in polynomial social relationships. Gregory and colleagues [5] presented soundless videos of two women socially interacting with college students and examined their gaze behavior. They divided the participants into socially engaged, non-engaged, and free-viewing groups. Then, they informed the socially engaged and the non-engaged groups that they would either interact with the two women in the video or not interact. They instructed the free-viewing group to watch the video only. The results of eye-tracking data indicated that the free-viewing group showed significantly more correct gaze-following behaviors and longer dwell times on faces than the other groups, whereas the other two groups observed the body areas longer than the face areas. These findings suggest that presenting a social context reduces visual attention and gaze-following behavior. Another study [6] compared the eye-movement patterns of dyadic and triadic social relationships in typically developing children (TDC) and children with autism spectrum disorders (ASD). The study collected eye-movement data from live conversational interactions with one or two people. The results showed that ASD children had more fixations in triadic than dyadic situations, whereas TDC showed the opposite eye-movement patterns with more fixations in dyadic than triadic social relationships. Both groups equally observed the other person’s face in dyadic and triadic settings in the baseline phase, and TDC participants showed slightly shorter latencies for observing the target face than ASD participants. These results suggest that people with communication difficulties, such as ASD have difficulty detecting faces containing social information in triadic and polynomial social relationships. However, these two studies [5,6] did not collect data on the non-observed stimuli. Tsai and colleagues [7] reported that typically developing adults had a longer total fixation duration on chosen than rejected stimuli and a longer total looking time at factors they should consider in responding to multiple-choice problems.

We must examine gaze-following behaviors and how people observe non-gaze-shifted faces and non-observed object stimuli to understand the effect of multiple visual cues. Moreover, humans are exposed to various auditory social cues in naturalistic settings, and polynomial social interactions require us to respond to the correct visual or auditory cues in complicated situations comparing with triadic social interactions. We mainly depend on seeing and hearing to perceive everyday external information. Therefore, we must also understand how auditory social context cues affect eye-movement patterns in polynomial social relationships. Dyadic and triadic relationships of infants rely on eye contact and eye gaze to visually acquire external information. However, children up to 6 to 7 years tend to respond to auditory stimuli more than visual stimuli [8,9]. Children aged 9-to-10 years show a transition. Children aged 12 years are more likely to respond to visual than auditory stimuli when a compound audio-visual stimulus is presented [9]. We also know that children find it more challenging to ignore auditory aspects of audio-visual stimuli [10,11]. Ross and colleagues [10] presented emotional body movements as visual stimuli, emotional spoken sounds as auditory stimuli, and presented combined audio-visual stimuli to children and adults. They demonstrated that children and adults could identify auditory stimuli when they presented mismatched visual stimuli, but children, compared to adults, found it more challenging to detect simultaneously presented visual stimuli incongruent with auditory stimuli. Another study [10] showed that children responded to auditory stimuli more often than visual stimuli when researchers simultaneously presented pictures of animals and congruent auditory stimuli of the animals to children. These findings [10,11] help us understand the preference for audio and visual stimuli in polynomial settings. However, we do not know how people observe the visual aspects of compound stimuli in polynomial social relationships. We also need to investigate the difficulties in ignoring auditory cues between children and adults by observing the first fixation latencies in detecting stimuli.

This study examined whether college students and typically developing children show similar gaze-following behavior under two triadic and polyadic conditions. We asked the participants to observe a target visual stimulus that was looked at by a face stimulus while we presented a spoken name that was congruent or incongruent with the target visual stimulus. We predicted that all the participants would show fewer fixations and shorter fixation durations in the polyadic compared to the triadic condition. We also expected a longer time to the first fixation for incongruent stimuli, especially in typically developing children than adults, because children often find it more challenging to ignore auditory stimuli [10,11].

## Materials and methods

### Participant

College students (CS; n = 24, 7 men and 17 women; Mean Age 24.17 years, SD = 7.34) and 19 typically developing children (TDC; 12 boys and 7 girls, Mean Age 11.30 years, SD = 3.17) participated in this study. Waseda University Institutional Review Board (IRB) permitted this work (approval number: 2021–152). After the IRB permission, we asked students and children living in Tokyo and the mid-west regions of Japan to participate in our research. CS participants gave their written informed consent before data collection and participation in this study. We explained the details of this study to TDC participants and their parents using written documents with oral explanation. After this explanation, the parents gave their written informed consent if they and the TDC participants agreed to participate in this study. We made the participants anonymous by using unrelated numbers. After the data collection, we accessed information that could identify individual participants using these numbers. None of the participants had a diagnosis of developmental disabilities before the study. All the TDC were enrolled in mainstream school classes.

### Apparatus and stimuli

We experimented with CS in a university laboratory and with TDC in a room close to their homes. We used a laptop computer (Dell, Precision 5540, Windows 10.1) to control the experiment. A display (Dell, P2419H, 1920×1080, 23.8 inches) was used to present the stimuli and to connect an eye tracker (Tobii, X3-120) used to measure the participants’ eye movements and software (Tobii, Tobii Pro Lab) used to analyze the eye-movement data. We drew five face stimuli with eyes oriented to the upper left, upper right, lower left, lower right, or with the eyes closed. Using royalty-free materials, we prepared 16 target visual stimuli in four categories representing animals (dog, cat, bird, and fish), foods (spaghetti, pizza, Hamburg steak, and rice omelet), sports (baseball, basketball, soccer, and skiing), and emotions (happy, angry, sad, and surprised) on the CD-ROM (Fan Font Alliance Network, SOZAI×FAN). Each category’s four target visual stimuli were presented on the display’s upper left, upper right, lower left, and lower right corners. We also prepared recordings of the spoken names of the 16 visual stimuli.

### Conditions

**Fig 1:**
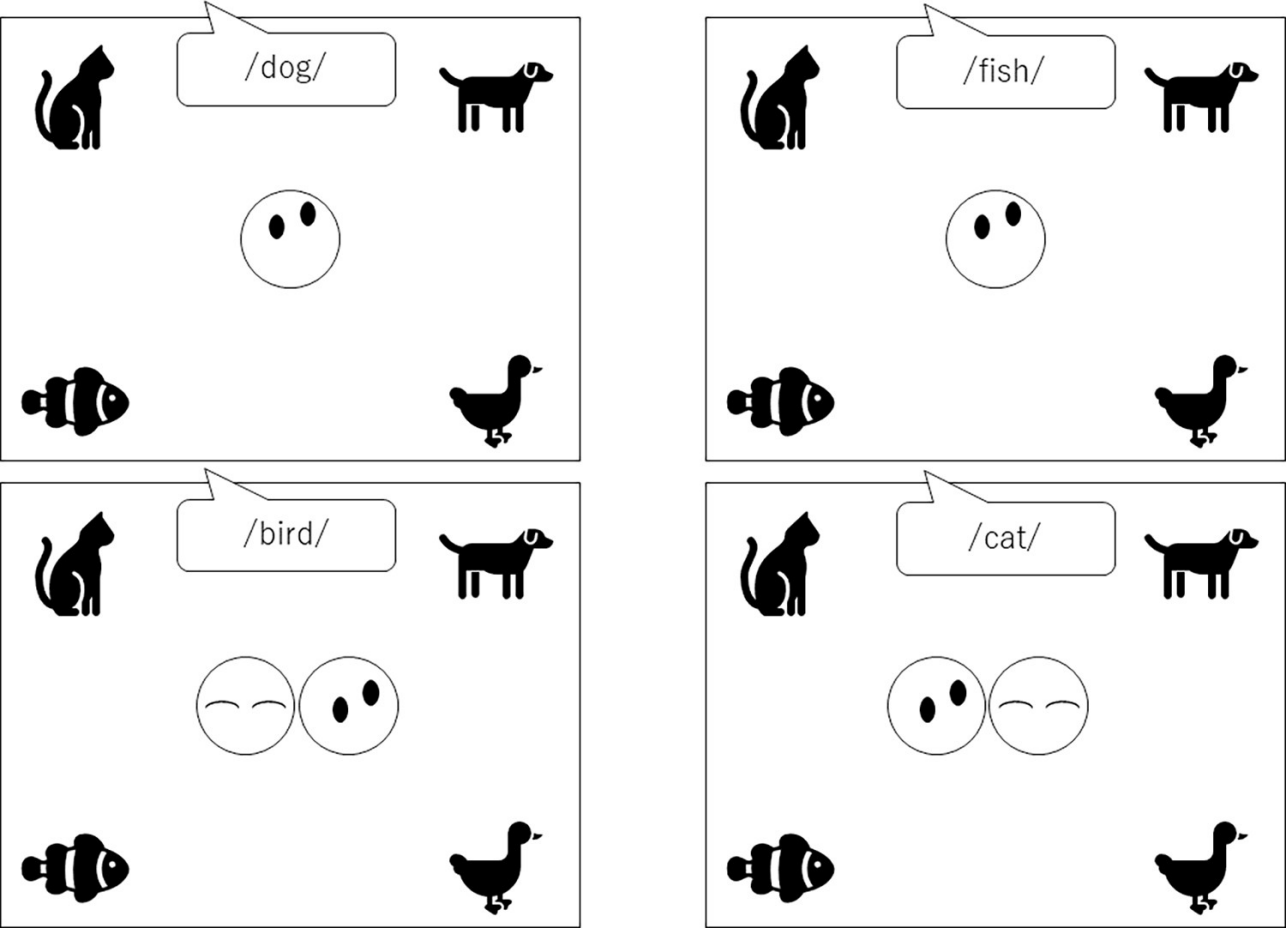
Two triadic and polyadic conditions. The top left panel shows the triadic, congruent condition, and the top right shows the triadic, incongruent condition. The bottom left panel shows the polyadic, congruent condition, and the bottom right panel shows the incongruent condition. In the polyadic condition, the two face stimuli were presented side-by-side in the middle of the display, and we randomized the positions of eyes-opened face and closed faces.

We presented face stimuli with the four eye orientations nine times, such that there were 36 trials in this experiment’s triadic and polyadic conditions. We presented the visual target stimuli and their spoken names congruently in 12 and incongruently in 24 trials, totaling 36 trials. Fig 1 shows sample stimuli in the triadic and polyadic conditions. In the triadic condition, one face stimulus was presented in the middle of the display, looking at one of the four visual stimuli on the corners of the display while listening to either the congruent spoken name (e.g., “dog” when the face was looking at a picture of a dog [top left]) or an incongruent name (e.g., “fish” when the face was looking at a picture of a dog [top right]). In the polyadic condition (bottom two panels), two face stimuli were presented side-by-side in the middle of the display and we randomized the positions of the eyes-opened and closed faces (48% on the left and 52% on the right face). One face stimulus had the eyes closed, and the other face stimulus was looking at one of the four visual stimuli on the corners of the display while listening to either the congruent spoken name (e.g., “bird” when the right face was looking at a picture of a bird [bottom left]) or an incongruent spoken name (e.g., “cat” when the left face was looking at a picture of a bird [bottom right]).

### Procedure

The participants sat approximately 70cm away from the display without using chinrest, and we instructed them to observe the nine red dots presented on the display for calibration. After completing the calibration, we instructed the participant to observe what the face stimulus looked at regardless of what they heard within 2 s. In a trial visual stimulus was presented for 2 s and auditory stimulus was presented only once for 500 ms, concurrently with the presentation of the visual stimulus. After visual-auditory stimulus presentation, a black blank slide was presented for 1 s and the next stimuli were presented. Participants started in either the triadic or the polyadic condition and conducted the other condition after finishing the first condition. We did not give the participants feedback about whether they were looking at the target stimuli. Most participants conducted 36 trials under each condition. However, the seven younger children (mean age = 8.42, SD = 1.63) conducted only 16 trials in which half were congruent and the other half were incongruent. In terms of data quality, all participants recorded 76.45% (SD = 11.80) of sampling rate. We thought the data quality were sufficient and we decided to use all data from them.

### Dependent variables and data analysis

Before data collection, we defined the software’s area of interest (AOI) in assessing the target observing behaviors. The data were collected with Tobii Pro Lab software using the Tobii Fixation filter. We defined the eyes-open faces, the four visual stimuli on the displays’ corners in the two conditions, and the eyes-closed stimuli in the polyadic condition as AOIs. The dependent variables were the fixation number, which indicated the frequency at which participants observed the stimuli in the AOIs. The mean fixation duration indicated the time a participant observed the stimuli in a single fixation. The time to the first fixation for each stimulus indicated the time taken to observe each stimulus in a trial for the first time. If the participants looked at the stimulus very quickly, the time to the first fixation was close to 0, whereas if the participant found it difficult to find the target stimulus, the time to the first fixation would be closer to 2 s. We believe that the fewer number of fixation and shorter fixation duration would show the difficulties in searching the target visual stimuli and in social communication [4,5]. Longer time to first fixation would reflect difficulties in communication and ignoring auditory aspect of audio-visual stimuli [6,10]. The higher interference rate would show the preference audio and visual stimuli in polynomial settings or multiple-choice questions [10,11].

We conducted mixed factorial analyses of variance (ANOVAs) on the number of fixations, the mean fixation duration, the time to the first fixation for each stimulus, and interference rate using a 2 (group: CS and TDC; between group) × 2 (face conditions: triadic and polyadic; within group) × 2 (congruency conditions: congruent and incongruent; within group) design to compare face type and congruency type effects on eye movement patterns in the two groups. We also conducted a 2 (group: CS and TDC; between group) × 2 (congruency conditions: congruent and incongruent; within group) ANOVA for the face stimuli interference rate because the triadic condition did not have an eye-closed face trial. We also conducted a mixed factorial ANOVAs on eye movements data using a 2 (group: CS and TDC; between group) × 2 (position conditions: same side and opposite side; within group) design to examine the face position effects of the target visual stimulus in polynomial conditions on the eye movement patterns of the two groups (see S1 file). We then conducted post hoc analyses using Bonferroni’s multiple comparison tests in necessity. If there were group differences between CS and TDC, we investigated how TDC’s chronological age affected these results. Previous study showed that children aged 12 years are more likely to respond to visual than auditory stimuli when a compound audio-visual stimulus is presented [9]. Therefore, we divided the TDC group into two groups, with those aged 12 and older as older group and the rest as younger group. We then conducted secondary analyses using ANOVA on the face and target stimuli as dependent variables using 2 (group: older and younger; between group) × 2 (face conditions: triadic and polyadic; within group) × 2 (congruency conditions: congruent and incongruent; within group) design to compare face type and congruency type effects on eye movement patterns in the two groups.

## Results

### Face stimuli

Table 1 shows the eye-movement patterns on the face stimuli. The number of fixations showed that CS fixated 1.23 times (SE = .09) and TDC fixated 1.26 times (SE = .15) on eyes-opened faces. There were significant main effects of the face [*F* (1, 41) = 26.53, *p* < .001, *η*^*2*^ = .39] and congruency [*F* (1, 41) = 4.15, *p* < .05, *η*^*2*^ = .09], which indicated that the participants observed the eyes-open face more often in the triadic than the polyadic condition and the congruent than in the incongruent condition. The mean fixation duration indicated that CS took 171.01 ms (SE = 9.01) and TDC took 158.97 ms (SE = 9.25) for a single fixation on the eyes-open face. Moreover, there was a significant main effect of the face [*F* (1, 41) = 31.32, *p* < .001, *η*^*2*^ = .43], which indicated that the participants observed the eyes-open face longer in the triadic than the polyadic condition. The time to the first fixation indicated that CS took 139.45 ms (SE = 20.00), and TDC took 276.75 ms (SE = 57.02) to fixate on the eyes-open face for the first time. There were significant interactions between the group and congruency [*F* (1, 41) = 10.13, *p* < .005, *η*^*2*^ = .20] and the face and congruency [*F* (1, 41) = 4.57, *p* < .05, *η*^*2*^ = .10]. Post-hoc analysis revealed simple main effects of the group and congruent conditions [*F* (1, 82) = 19.63, *p* < .001, *r =* .44], and the congruency factor and TDC participants [*F* (1, 82) = 12.04, *p* < .001, *r* = .36]. These results indicated that CS participants fixated faster on eyes-open faces than TDC participants, and TDC participants took longer to fixate on the eyes-open face in the congruent than incongruent condition. There were also other simple main effects of the face and congruent [*F* (1, 82) = 21.71, *p* < .001, *r* = .46] and incongruent conditions [*F* (1, 82) = 6.22, *p* < .05, *r* = .27], and of the congruency factor and the TDC group [*F* (1, 82) = 7.41, *p* < .01, *r* = .29]. These results revealed that the participants fixated faster on the eyes-open face in the triadic than the polyadic condition under congruent and incongruent conditions. The participants took longer to fixate on the face stimulus in the polyadic, congruent condition than in the incongruent condition. The interference rate results indicated that the mean percentage of non-target face stimuli was 28.54% (SE = .02) in CS and 36.68% in TDC (SE = .05). Moreover, there was a significant main effect of the group [*F* (1, 41) = 9.87, *p* < .001, *η*^*2*^ = .09] indicating that TDC participants observed the eyes-closed face more often than the CS participants.

**Table 1:** Eye movement patterns on face stimuli.

Dependent variables	Groups	College students		children		ANOVA	
	congruency	triadic	Poly nominal	Triadic	Poly nominal	main effect	interaction
Number of fixations (#)	congruent	1.33 (0.08)	1.11 (0.12)	1.45 (0.18)	0.97 (0.12)	Face: *F* (1, 41) = 26.53, *p* < .001**** Congruency: *F* (1, 41) = 4.15, *p* < .05*	*NS*
	incongruent	1.40 (0.07)	1.09 (0.09)	1.63 (0.17)	1.01 (0.14)		
Mean fixation duration (ms)	congruent	203.22 (10.93)	147.93 (10.64)	173.43 (10.30)	143.21 (8.54)	Face: *F* (1, 41) = 31.32, *p* < .001****	*NS*
	incongruent	190.45 (9.12)	142.44 (5.34)	175.33 (10.86)	143.92 (7.30)		
Time to first fixation (ms)	congruent	44.27 (11.48)	207.00 (22.31)	233.36 (77.40)	415.49 (59.65)	Group: *F* (1, 41) = 11.46, *p* < .005*** Face: *F* (1, 41) = 17.21, *p* < .001****	Group× Congruency: *F* (1, 41) = 10.13, *p* < .005*** Face× Congruency: *F* (1, 41) = 4.57, *p* < .05*
	incongruent	84.68 (31.74)	221.85 (14.45)	205.03 (61.30)	253.10 (29.73)		
Interference rate (%)	congruent	-	27.41% (0.03)	-	39.50% (0.05)	Group: *F* (1, 41) = 9.87, *p* < .005***	*NS*
	incongruent	-	29.68% (0.02)	-	37.86% (0.06)		

Numbers in parentheses represent standard errors. NS = not significant.

### Target visual stimuli

Table 2 shows the eye-movement patterns on the target visual stimuli. The number of fixations indicated that CS fixated 3.43 times (SE = .32) and TDC fixated 2.61 times (SE = .29) on the target visual stimulus. There were significant main effects of the group [*F* (1, 41) = 4.13, *p* < .05, *η*^*2*^ = .09] and the face [*F* (1, 41) = 7.57, *p* < .01, *η*^*2*^ = .16], indicating that CS participants observed the target stimuli more often than TDC participants. Moreover, all the participants observed the stimuli more often in the triadic than in the polyadic condition. Furthermore, the mean fixation durations showed that CS took 316.68 ms (SE = 32.30) and TDC took 285.32 ms (SE = 37.62) for a single fixation on the target stimulus. There were no significant main effects or interactions in the mean fixation duration on the target visual stimuli. Furthermore, the time to the first fixation indicated that CS took 784.50 ms (SE = 68.10) and TDC took 873.70 ms (SE = 80.69) to fixate on the target stimulus for the first time. There was a three-way interaction [*F* (1, 41) = 6.01, *p* < .05, *η*^*2*^ = .13], and post-hoc simple・simple analysis revealed significant interactions for group and congruency in the triadic condition [*F* (1, 82) = 4.66, *p* < .05, *r* = .23] and for face and congruency in the TDC group [*F* (1, 41) = 10.68, *p* < .005, *r* = .46]. Follow-up simple・simple analyses revealed significant main effects of the face in congruent [*F* (1, 82) = 11.15, *p* < .005, *r* = .35] and incongruent conditions [*F* (1, 82) = 12.88, *p* < .001, *r* = .37] for CS; in the congruent condition for TDC [*F* (1, 82) = 25.09, *p* < .001, *r* = .48], and the congruency factor in the triadic condition for TDC [*F* (1, 82) = 10.60, *p* < .005, *r* = .34]. These results suggested that CS fixated faster on the target visual stimulus in the triadic than the polyadic condition in congruent and incongruent conditions. In contrast, TDC fixated faster on the target stimulus in the triadic, congruent condition than in the polyadic condition. We also found that TDC participants fixated faster on the target stimulus in the triadic, congruent condition than in the incongruent condition. In contrast, the time to the first fixation on the target visual stimulus in TDC was similar in the polyadic, congruent, and incongruent conditions. Also, the interference rate indicated that the mean percentages of non-target face stimuli for CS were 1.72% (SE = .01) and 6.18% for TDC (SE = .02). There was a significant interaction between face and congruency [*F* (1, 41) = 5.51, *p* < .05, *η*^*2*^ = .12]. Post-hoc analysis revealed a simple main effect of the congruency factor in the triadic condition [*F* (1, 82) = 9.69, *p* < .005, *r* = .33]. These results suggested that the participants observed the non-target stimuli more often in the triadic, incongruent condition than in the congruent condition. However, they observed the non-target stimuli equally often in the polyadic condition.

**Table 2:** Eye movement patterns on target visual stimuli.

Dependent variables	Groups	College students		children		ANOVA	
	congruency	triadic	Poly nominal	Triadic	Poly nominal	main effect	interaction
Number of fixations (#)	congruent	3.73 (0.34)	3.30 (0.31)	2.86 (0.32)	2.47 (0.27)	Group: *F* (1, 41) = 4.13, *p* < .05 Face: *F* (1, 41) = 7.57, *p* < .01**	*NS*
	incongruent	3.60 (0.31)	3.10 (0.29)	2.60 (0.30)	2.53 (0.27)		
Mean fixation duration (ms)	congruent	330.85 (32.72)	299.19 (31.42)	279.81 (35.71)	268.78 (34.37)	*NS*	*NS*
	incongruent	320.59 (34.80)	316.11 (30.27)	278.27 (35.24)	314.42 (45.15)		
Time to first fixation (ms)	congruent	673.67 (67.45)	876.47 (71.20)	699.27 (50.93)	1001.95 (83.80)	Face: *F* (1, 41) = 34.12, *p* < .001****	Face× Congruency: *F* (1, 41) = 4.71, *p* < .05* Group× Face× Congruency: *F* (1, 41) = 6.01, *p* < .05*
	incongruent	684.83 (55.60)	903.04 (78.15)	869.98 (115.10)	923.59 (72.92)		
Interference rate (%)	congruent	0.66% (0.00)	0.32% (0.00)	2.87% (0.01)	6.75% (0.03)	Group: *F* (1, 41) = 7.86, *p* < .01** Congruency: *F* (1, 41) = 4.40, *p* < .05*	Face× Congruency: *F* (1, 41) = 5.51, *p* < .05*
	incongruent	4.73% (0.03)	1.18% (0.00)	8.81% (0.02)	6.29% (0.01)		

Numbers in parentheses represent standard errors. NS refers as not significant.

#### Secondary analysis on face stimuli in children

We found group differences in the number of fixations on the target stimuli, the time to first fixation on the target stimuli, and the interference rate in both face and target stimuli. However, we still needed to investigate how TDC’s age affected performance. Mean age of younger TDC was 8.97 (SD = 1.77) and of older TDC was 14.50 (SD = 1.59). Table 3 shows the eye-movement patterns on the face stimuli. The number of fixations showed that older TDC fixated 1.28 times (SE = .22) and younger TDC fixated 1.25 times (SE = .22) on eyes-opened faces. There was a significant interaction among the age, face, and congruency factors in the number of fixations [*F* (1, 17) = 6.42, *p* < .05, *η*^*2*^ = .27]. Post-hoc analysis revealed simple・simple main effects of the face factor of congruent condition in older TDC [*F* (1, 34) = 8.50, *p* < .01, *r =* .45], of the face factor of incongruent condition in younger TDC [*F* (1, 34) = 12.63, *p* < .005, *r =* .52], and of congruency factor on triadic condition in younger TDC [*F* (1, 34) = 8.34, *p* < .01, *r =* .44]. These results showed that older TDC participants fixated on the eyes-opened face more often in triadic than in polynomial congruent conditions whereas younger TDC participants more frequently fixated on the face in polynomial incongruent conditions. The mean fixation duration indicated that older TDC took 167.01 ms (SE = 13.58) and younger TDC took 153.13 ms (SE = 11.89). We also found a significant interaction among three factors [*F* (1, 17) = 4.68, *p* < .05, *η*^*2*^ = .22], showing the simple・simple main effects on the face factor of incongruent condition in older TDC [*F* (1, 34) = 9.70, *p* < .005, *r =* .47] and of congruent condition in younger TDC [*F* (1, 34) = 5.34, *p* < .005, *r =* .37]. These results showed that older TDC participants showed shorter fixation duration in polynomial incongruent condition than in triadic whereas younger TDC fixated shorter in those of congruent conditions. The time to the first fixation indicated that older TDC took 209.32 ms (SE = 47.25), and younger TDC took 325.79 ms (SE = 90.20) to fixate on the eyes-open face for the first time. We found a main effect in congruency factor [*F* (1, 17) = 7.11, *p* < .05, *η*^*2*^ = .30], which indicating that TDC participants could find the eye-opened face faster in incongruent condition than in congruent condition. The interference rate was 24.98% (SE = .05) in older TDC and 48.65% in TDC (SE = .07). Moreover, there was a significant main effect of the age [*F* (1, 17) = 7.11, *p* < .05, *η*^*2*^ = .30] indicating that younger TDC participants observed the eyes-closed face more often than the older participants.

**Table 3:** Eye movement patterns on face stimuli in children.

Dependent variables	Groups	Older TDC		Younger TDC		ANOVA	
	congruency	triadic	Poly nominal	Triadic	Poly nominal	main effect	interaction
Number of fixations (#)	congruent	1.57 (0.30)	0.94 (0.16)	1.36 (0.22)	0.99 (0.19)	Face: *F* (1, 17) = 15.04, *p* < .005***	Age×Face× Congruency: *F* (1, 17) = 6.42, *p* < .05*
	incongruent	1.51 (0.21)	1.11 (0.19)	1.72 (0.26)	0.94 (0.21)		
Mean fixation duration (ms)	congruent	174.59 (18.07)	155.82 (8.36)	172.59 (12.80)	134.04 (13.08)	Face: *F* (1, 17) = 10.05, *p* < .01**	Age×Face× Congruency: *F* (1, 17) = 4.68, *p <* .05*
	incongruent	194.80 (20.93)	142.83 (6.95)	161.17 (9.80)	144.70 (11.88)		
Time to first fixation (ms)	congruent	156.23 (84.24)	345.51 (53.20)	289.45 (119.43)	459.84 (95.68)	Congruency: *F* (1, 17) = 7.11, *p* < .05*	*NS*
	incongruent	76.00 (31.74)	250.53 (14.45)	298.87 (95.49)	254.97 (50.20)		
Interference rate (%)	congruent	-	23.57% (0.05)	-	51.09% (0.06)	Age: *F* (1, 17) = 7.11, *p* < .05*	*NS*
	incongruent	-	26.39% (0.05)	-	46.21% (0.09)		

Numbers in parentheses represent standard errors. TDC = typically developing children, NS = not significant.

#### Secondary analysis on visual target stimuli in children

Table 4 shows the eye-movement patterns on the target visual stimuli in children. The number of fixations indicated that older TDC fixated 3.01 times (SE = .36) and younger TDC fixated 2.33 times (SE = .42) on the target visual stimulus. However, there were no significant main effects and interactions. The mean fixation duration indicated that older TDC spent 331.00 ms (SE = 63.82) and younger TDC took 252.10 ms (SE = 43.01). We also found a significant interaction among three factors [*F* (1, 17) = 5.18, *p* < .05, *η*^*2*^ = .23], showing the simple・simple main effect tendency on the congruency factor of polynomial condition in younger TDC [*F* (1, 34) = 2.98, *p* <. 10^+^, *r =* .28]. The time to the first fixation indicated that older TDC took 732.06 ms (SE = 75.60), and younger TDC took 1068.45 ms (SE = 118.05) to fixate on the eyes-open face for the first time. We found main effects in age factor [*F* (1, 17) = 5.40, *p* < .05, *η*^*2*^ = .24] and face factor [*F* (1, 17) = 8.20, *p* < .05, *η*^*2*^ = .31], which indicating that younger TDC participants required longer latencies of looking at the target stimuli than older TDC and all participants took longer time to first fixation duration. Also, the interference rate indicated that the mean percentages of non-target visual stimuli for older TDC were 2.91% (SE = .01) and 8.56% for TDC (SE = .03). There was a significant interaction among three factors [*F* (1,17) = 5.36, *p* < .05, *η*^*2*^ = .24], indicating the simple・simple main effects on the age factor of polynomial congruent condition [*F* (1, 68) = 4.59, *p* < .05, *r =* .25] and on the congruency factor of triadic condition in younger TDC [*F* (1, 34) = 5.77, *p* < .05, *r =* .38]. These results showed that older TDC participants showed lower interference rate in polynomial congruent condition than younger participants, and younger TDC showed higher interference rate in triadic incongruent condition than in congruent condition.

**Table 4:** Eye movement patterns on visual target stimuli in children.

Dependent variables	Groups	Older TDC		Younger TDC		ANOVA	
	congruency	triadic	Poly nominal	Triadic	Poly nominal	main effect	interaction
Number of fixations (#)	congruent	3.46 (0.39)	2.91 (0.33)	2.42 (0.44)	2.16 (0.38)	*NS*	*NS*
	incongruent	2.99 (0.36)	2.67 (0.35)	2.31 (0.45)	2.43 (0.40)		
Mean fixation duration (ms)	congruent	290.22 (47.81)	339.07 (65.81)	272.24 (52.80)	217.65 (29.12)	*NS*	Age×Face× Congruency: *F* (1, 17) = 5.81, *p <* .05*
	incongruent	344.57 (66.09)	350.14 (75.59)	230.06 (32.95)	288.44 (57.18)		
Time to first fixation (ms)	congruent	598.20 (68.48)	806.20 (77.37)	772.78 (66.28)	1144.31 (118.21)	Age: *F* (1, 17) = 5.40, *p* < .05* Face: *F* (1, 17) = 8.20, *p* < .05*	*NS*
	incongruent	657.93 (78.40)	761.40 (88.24)	1024.20 (180.02)	1041.55 (96.26)		
Interference rate (%)	congruent	1.34% (0.01)	1.55% (0.01)	3.97% (0.01)	10.53% (0.05)	*NS*	Age×Face× Congruency: *F* (1, 17) = 5.36, *p <* .05*
	incongruent	4.26% (0.01)	4.49% (0.01)	12.11% (0.05)	7.60% (0.02)		

Numbers in parentheses represent standard errors. TDC = typically developing children, NS = not significant.

## Discussion

We examined whether CS and TDC participants showed similar gaze-following behaviors under triadic and polyadic conditions. As we predicted, all the participants showed fewer fixations on the eyes-opened face and target object stimuli under the polyadic than the triadic condition. Moreover, we found a shorter fixation duration on the eye-opened face stimulus only in the polyadic condition, whereas participants showed comparable fixation durations on the target stimuli under both conditions. A previous study [6] indicated that TDC show fewer fixations in triadic than in dyadic social relationships. However, McParland et al. did not discuss the mean fixation duration across the two conditions and eye-movement patterns between adults and children. The current study’s results extended the previous finding [6] that increasing people in social relationships decreases adults’ and children’s fixations on target faces and object stimuli, resulting in a shorter mean fixation duration on the face stimulus.

We did not find significant differences in face stimuli observations between CS and TDC participants. Previous studies show that adult participants [3,4] and those with fewer communication skills [6] displayed longer saccade latencies and made more fixation errors in detecting eye gaze locations when there were over two people and an unexpected person presented visual cues. Therefore, we expected TDC to show longer latencies and more fixation errors on the target stimuli, reflecting their communication difficulties with other people [3,6]. However, the results showed that CS and TDC participants took longer for the first fixation on faces in polynomial than triadic relationships with no group differences. We also found that TDC participants were more distracted when observing the eyes-open face than CS participants. These results suggest that presenting multiple faces does not delay detecting visual social cues, but children observe unrelated stimuli more often when seeing multiple faces.

Previous studies [10,11] have shown that presenting compound audio-visual stimuli leads adults and children to make more visual than auditory errors. The current study’s participants showed more fixations in the incongruent than the congruent condition. Therefore, we expected them to show more visual errors, especially in incongruent conditions. However, the results for the congruent condition were opposite to our expectations. Wille and Ebersbach [11] showed more visual errors in the congruent condition when object stimuli, such as a cow and its mooing sound, were presented. However, Ross and colleagues [10] reported more visual errors in incongruent conditions when they presented an image of a body and the related emotional sounds. Stimulus presentation differences could cause these differences between studies. For example, Wille and Ebersbach [11] instructed participants to detect whether a stimulus they presented was visual-only, auditory-only, or bimodal without telling the participants what they must ignore. In contrast, Ross and colleagues [10] asked the participants to detect emotions based on the instruction to ignore specific stimuli. We also instructed participants to look at the stimuli observed by the face while ignoring the auditory stimuli, which might have made it more challenging to detect visual stimuli in incongruent conditions while ignoring auditory aspects of compound audio-visual stimuli.

Based on the above, we expected that CS and TDC participants would show shorter latencies for observing the face stimuli in the congruent than in the incongruent condition. However, TDC participants unexpectedly showed a longer time to the first fixation on faces in the congruent than in the incongruent condition. The CS participants constantly relocated their fixation in the middle of the display prior to the stimulus presentation. We presented the face stimulus in the middle of the display, especially in the triadic condition, so that CS participants would notice that the face always appeared in the exact location, whereas TDC participants would not. In the polynomial conditions, participants had to search for the eyes-open face when two faces were presented, which increased the chances of TDC participants observing the face stimuli. It would also explain why there was no difference between the groups in the incongruent condition. The results indicated that TDC participants ignored the face stimuli in the congruent condition because they could search for the target stimuli without observing the eye direction. When deciding which modality to use in the early observation stage, children might get confused. Ross and colleagues [10] showed that child participants responded more accurately in the congruent than the incongruent condition. They also showed the highest accuracy when we presented an auditory stimulus alone. These results suggest that although we instructed the participants to observe the visual stimulus in compound stimuli, child participants tried to respond to the auditory aspects of the compound stimulus. Therefore, the fixation latencies on children’s faces in the congruent condition were longer.

Previous studies [8–10] have shown that children aged 11-to-12 responded to visual stimuli more often than auditory stimuli whereas children from 6-to-7 years tended to respond to auditory stimuli more often. Compared with previous studies [8–10], our TDC participants varied in age ranging from 6.6 years old to 17.6 years old. Therefore, we wanted to investigate the age effects by dividing into two groups based on age 12: older TDC and younger TDC. Our results showed that TDC’s chronological age affected the eye movements toward visual target stimuli. In other words, younger TDC were more sensitive to the auditory stimuli than visual stimuli by showing longer latencies to the visual target stimulus than older TDC. Whereas previous studies did not collect eye movement data [8–10], our data suggest that we can index the shift to visual dominance in children from a lower to a higher age using an eye-tracking system. It was also important to note that younger TDC participants showed auditory dominance in triadic conditions showing higher interference rates on incongruent conditions while interference rates in polynomial conditions were similar. Moreover, younger TDC showed higher interference rate in polynomial congruent condition than older TDC. Previous studies showed the auditory dominance of younger TDC in dyadic conditions [8–10] and children were more likely to respond to auditory stimuli of audio-visual congruent stimuli [10,11]. However, our results extend the previous findings [8–11] that younger TDC showed auditory dominance in triadic conditions and that younger than 12 years old TDC were more sensitive to auditory stimuli in audio-visual stimuli in polynomial congruent condition whereas older TDC showed visual dominancy. Moreover, whereas previous studies only showed the development and transition of audio-visual integration in dyadic interactions showing the visual dominance in older TDC known as the Colavita effect and the auditory dominance known as reversed Colavita effect [8–11], we found the developmental change of sensory dominance in triadic and polynomial social interactions for children around 12 years old.

CS participants showed more eye movement fixations on the target object stimuli than TDC participants, and both groups showed more fixations in the triadic than the polynomial condition, whereas there were no differences in the mean fixation duration. Tsai and colleagues [7] reported that adult participants showed longer total fixations on chosen stimuli and factors determining the responses to multiple-choice problems. This study did not collect data on the total fixation duration. However, we could calculate it by multiplying the fixation count and mean fixation duration, which replicated Tsai and colleagues’ findings of a high fixation count for the chosen target stimuli, with no difference in the mean fixation durations. Dalmaso and colleagues [3] reported that typically developing adults had longer saccade latencies and made more saccade direction errors in triadic social relationships when they followed a person’s gaze to visual cues inconsistent with where the face stimulus was looking than when it was consistent. We did not collect information on gaze direction, but child participants showed higher interference rates for correct stimuli than adult participants. Moreover, both participants showed higher interference rates in the incongruent, triadic than in the congruent, triadic condition, which might reflect gaze direction errors for incongruent stimuli [3].

This study also extended the findings of Dalmaso and colleagues [3], showing that inconsistent audio-visual stimuli result in longer fixation latencies in adult participants for the correct stimuli in triadic and polynomial conditions, and longer latencies in child participants for triadic conditions. We expected that child participants in more complicated settings would clearly show longer latencies and more errors in the incongruent polynomial conditions than in the congruent condition [3,4]. However, child participants did not show a difference in latencies or errors in the polynomial condition with considering age effects, possibly because of simultaneous discrimination or successive discrimination difficulties [12,13]. Children often incorrectly respond when choosing the correct stimulus from a series of comparison stimuli when the corresponding sample stimulus is presented [12,13] in typical conditional discrimination tasks. The tasks of the current study are mixtures of simultaneous discrimination tasks for selecting the correct comparison stimulus among alternatives while conducting successive discriminations of alternatives containing sample and comparison stimuli. Children in the current study were required to detect eyes-open faces and the location of a target stimulus through simultaneous discrimination, and then in the polynomial condition, they were required to detect the gaze-directed stimuli through successive discrimination. In contrast, we did not require the child participants to detect the eyes-open face stimulus in the triadic condition. We also presented auditory stimuli. Therefore, presenting too many visual stimuli might cancel audio-visual inconsistencies in child participants for the polynomial condition. Contrary, older and younger TDC did show the sensory dominances of audio-visual stimuli in polynomial congruent condition. These results could also suggest that younger child participants in the current study were auditorily dominant in triadic social relationships, but older TDC were visually dominant in polynomial social relationships. Future research should investigate how and when audio-visual dominances would divide in polynomial incongruent condition.

There are several limitations to this study. Firstly, unlike previous studies [8–11], this study did not require participants to detect the target stimuli behaviorally, such as by pressing a button. Instead, we collected the interference rates from an eye-tracking system, even though we could have also collected the error rates from behavioral data. In addition, we presented stimuli only for 2 s in each trial which might be too short to detect the target stimuli especially in polynomial conditions. It is possible that a greater number of visual stimuli in the same duration would decrease the opportunities of observing each stimulus than fewer number of stimuli. Therefore, future research should include behavioral response data to detect what participants have observed, or they should increase the presentation duration. We would also like to prolong the stimulus presentation duration of polynomial condition to ensure the observing opportunities. Secondly, although we conducted ANOVAs with age factors, we still needed to collect the eye-movement data of age related TDC participants. Chronological age is a critical for investigating response preferences to auditory and visual stimuli [8–11] in this study. However, we could only prepare two groups of children older than 12 and younger than 12. Therefore, for example, future research might include a TDC group of 7-year-olds, 9-year-olds, and older than 12-year-olds. In addition, children are less communicative than adults. Nevertheless, we did not collect descriptive data on participants’ communication abilities. We suggest that future research objectively compare the participants’ communication skills, for example, by using a standardized questionnaire. Thirdly, we used pictures of face stimuli to present the gaze cues to cancel out the face movement towards the target visual stimuli. However, most previous studies [8–11] used real human faces or movements. These differences in stimuli might have affect the results of this study. Moreover, how children communicate in triadic and polynomial conditions in naturalistic settings remains unknown. Therefore, we suggest using actual face stimuli in the polynomial conditions in future research. We believe using actual face and object stimuli would make our procedure more naturalistic setting than using picture stimuli. Finally, including a group of ASD participants would allow us to analyze the development and learning of social interactions in polynomial social relationships. McParland and colleagues [6] reported that appropriate training could change gaze behaviors. It is well-known that acquiring triadic social relationships lead to the development of language and social communication [1,2]. However, specific people often struggle to communicate in multi-person settings [4–6]. Knowing the children’s transition from triadic to polynomial social relationships would help us understand the different language and social communication development paths. Therefore, we plan to develop training methods for improving polynomial social relationships of children with communication difficulties and investigate whether eye movement patterns change with behavioral improvements.

## Supporting information

S1 File Analyzing the face position effect. (PDF)

S2 File Raw data. (XLSX)
