# Supplementary material for: Why is it difficult for children and adults to follow a person’s eye gaze in polynomial social relationships with compound audio-visual stimuli: An eye-tracking study
Source: PLoS One. 2023 Aug 1;18(8):e0289404. doi: 10.1371/journal.pone.0289404 (PMC10393156; doi:10.1371/journal.pone.0289404)
Supplement: S1 File — (PDF) [file pone.0289404.s001.pdf]

Analyzing the face position effect

We randomized the position of eyes-opened and eyes-closed faces presented side-by-side. However, we still needed to investigate how the face position affected the eye movement during gaze following. In other words, we needed to demonstrate different eye movement patterns when the target visual stimulus was presented on the same side as the eyes-opened face (e.g., left face with target visual stimuli on left) compared to when the target stimulus was presented on the opposite side to the eyes-opened face (e.g., left face with target visual stimuli on right). Therefore, we conducted mixed factorial ANOVAs on each type of eye movement data using a 2 (group: CS and TDC; between group) × 2 (position conditions: same side and opposite side; within group) design to examine the face position effects of target visual stimulus in the polynomial conditions on eye movement patterns of the two groups. The results showed main effects of the group factor on the interference rate [F (1, 41) = 4.16, p <.05, η^2 =.10] and of the face position factor on the time to first fixation [F (1, 41) = 5.01, p <.05, η^2 =.11]. These results indicated that the TDC participants showed a higher interference rate than CS participants (6.11% vs. 0.42%), and that all participants took longer to fixate on stimuli presented on the same side as the eyes-opened face than on the opposite side to the eyes-opened face (971.70 ms VS 892.03ms). We also found a significant interaction in the number of fixations [F (1, 41) = 7.03, p <.05, η^2 =.15]. Post hoc analysis revealed a simple main effect of the group and the opposite position conditions [F (1, 82) = 4.97, p <.05, r=.24], showing that TDC participants fixated

less than CS participants when the target stimulus was presented on the opposite side to the eyes-opened face (2.41 vs 3.35). Moreover, there was a simple main effect in the face position of the TDC group [F (1, 41) = 5.69, p <.05, r=.35], indicating that TDC participants fixated less when the target stimulus was presented on the opposite side of the eyes-opened face than on the same side (2.41 vs. 2.75).
